# Neurophysiological differentiation of upper motor neuron damage in neurodegenerative disorders

**DOI:** 10.1016/j.cnp.2022.09.002

**Published:** 2022-09-23

**Authors:** Yuichiro Shirota, Juuri Otsuka, Tatsushi Toda, Masashi Hamada

**Affiliations:** a Department of Neurology, Graduate School of Medicine, The University of Tokyo, 7-3-1 Hongo, Bunkyo-ku, Tokyo 113-8655, Japan; b Department of Clinical Laboratory, The University of Tokyo Hospital, 7-3-1 Hongo, Bunkyo-ku, Tokyo 113-8655, Japan

**Keywords:** AH, Abductor halluces, APB, Abductor pollicis brevis, ALS, Amyotrophic lateral sclerosis, ANOVA, analysis of variance, CMAP, compound muscle action potential, CMCT, central motor conduction time, EMG, electromyography, FDI, first dorsal interosseous, LMN, lower motor neuron, MEP, motor evoked potential, MSA, multiple system atrophy, M1, primary motor area, TA, tibialis anterior, TMS, transcranial magnetic stimulation, UMN, upper motor neuron, Amyotrophic lateral sclerosis, Central motor conduction time, Motor evoked potential, Multiple system atrophy, Neurophysiology, Pyramidal tract

## Abstract

**Objective:**

Using transcranial magnetic stimulation (TMS) to delineate upper motor neuron (UMN) signs of two neurodegenerative disorders: amyotrophic lateral sclerosis (ALS) and multiple system atrophy (MSA).

**Methods:**

Medical records including clinical signs for UMN damage and TMS results were reviewed retrospectively. The UMN signs were classified into none, mild, and severe based on neurological examination of various reflexes. Then TMS-elicited motor evoked potentials (MEPs) were recorded from a hand and a leg muscle to calculate the central motor conduction time (CMCT), which represents fast, mono-synaptic conduction along the corticospinal tract. Relations between the UMN signs and CMCT were analysed for the two diseases.

**Results:**

Prevalence and severity of the UMN signs for ALS and MSA were comparable for both upper and lower limbs. However, abnormality in CMCT was found more frequently in ALS: CMCT abnormalities were found in upper limbs for 44% in ALS patients but only for 7% in MSA patients; CMCT abnormalities in lower limbs were 55% in ALS and 20% in MSA. Some ALS patients showed abnormal CMCT in limbs without UMN signs, which was not true for most MSA patients.

**Conclusions:**

The abnormalities of CMCT were different in ALS and MSA, even for those who clinically had similar UMN signs. Sometimes, CMCT can reveal UMN damage in the absence of clinical UMN signs. Differences presumably derive from selective degeneration of different fibres in the motor descending pathways. Longitudinal studies must be conducted to accumulate neuroimaging and pathological findings.

**Significance:**

CMCT can be useful to differentiate ALS and MSA.

## Highlights

• ALS and MSA presented with similar profiles of upper motor neuron signs.
• Central motor conduction time was more abnormal in ALS than in MSA.
• Different structures may be involved in ALS and MSA along the corticospinal tract.

## 1 Introduction

Upper motor neuron (UMN) signs, or so-called pyramidal signs, are cardinal features of many neurological disorders. The UMN signs include Babinski sign, exaggerated tendon reflex, and spasticity, primarily caused by lesions in the primary motor area (M1) and its descending pathways. Although the signs are quite familiar to many neurologists, no precise pathophysiology underlying them is known (Lance, 2002, van Gijn, 1996).

Transcranial magnetic stimulation (TMS) facilitates investigation of motor descending pathways in a non-invasive manner. A TMS coil placed over the skull produces electric fields in the brain through electromagnetic induction. When the fields activate neurons in the M1 sufficiently, action potentials travel down the descending motor pathways, eventually giving rise to muscle twitch. Electromyography (EMG) corresponding to the twitch is termed motor evoked potential (MEP). Because MEP is believed to represent fast, monosynaptic conductions from the M1 to the spinal cord, central motor conduction time (CMCT) estimated from the MEP latency can be used as an index for conduction along the motor descending pathways. For example, a lesion such as a plaque of multiple sclerosis in the spinal cord would prolong CMCT, indicative of demyelinating characteristics of the lesion (Schlaeger et al., 2013). Nevertheless, little evidence is available to specify whether CMCT is abnormal along with clinical UMN signs for different neurological disorders.

To elucidate relations between TMS measures including CMCT and UMN signs in different neurodegenerative diseases, we specifically examined data of patients with amyotrophic lateral sclerosis (ALS) and multiple system atrophy (MSA). First, ALS and MSA present with the UMN signs in a large proportion of patients. Reports of the literature describe that 40–70% of ALS patients (Gubbay et al., 1985, Zoccolella et al., 2006) and 7–73% of MSA patients (Köllensperger et al., 2010) show UMN signs. Second, pathological studies have revealed that different fibres of the corticospinal tract are affected in ALS and MSA. Large, myelinated fibres are more adversely affected by damage in ALS, whereas smaller myelinated fibres undergo marked degeneration in MSA (Sobue et al., 1987). Given that large fibres have fast conduction velocity, in principle, we inferred that CMCT testing, mainly indicating conduction time along the fast fibres, might be more abnormal in ALS than MSA. As well as such pathological differences, the major diagnostic criteria for MSA (Gilman et al., 2008) indicate that “Babinski sign with hyperreflexia” is an additional feature; therefore MSA was considered suitable as a comparator for ALS in this study. Third, evidence is scarce in support of more detailed differentiation, including whether some symptoms are more frequent, or whether CMCT is useful to compare UMN damage associated with ALS and MSA.

This retrospective study is aimed at exploring potential association between the UMN signs and CMCT testing in ALS and MSA. In addition to comparison of CMCT, we assessed correlation between CMCT and UMN signs for both diseases.

## 2 Material and methods

### 2.1 Study approval and patients

This study is a retrospective, observational study. The study protocol was approved by the ethics committee of the Faculty of Medicine of the University of Tokyo. Medical records were inspected from 2015 to 2019 for ALS patients, and from 2009 to 2019 for MSA patients to obtain similar numbers of records for both. Inclusion criteria were possible, probable or definite ALS according to the revised El Escorial criteria (Brooks et al., 2000), and possible or probable MSA with cerebellar ataxia (MSA-C) or MSA with predominant parkinsonism (MSA-P) by Gilman’s criteria (Gilman et al., 2008). In brief, the criteria require that probable MSA should be a sporadic, progressive, adult-onset disease characterized by autonomic failure together with poorly levodopa-responsive parkinsonism or a cerebellar syndrome. Combination of suggestive features (including parkinsonism and cerebellar syndromes) and less severe autonomic dysfunction would fulfil the criteria for possible MSA.

### 2.2 Clinical information

Clinical information was collected with some emphasis on the UMN signs. Age, height, and duration from onset to CMCT testing were extracted from the records. As a measure of activity of daily living, the Barthel Index was extracted whenever possible.

The UMN signs were represented as the UMN score based on an earlier report (Quinn et al., 2020). For the upper limbs, each of the following was counted as one point: exaggerated biceps reflex, brachioradialis reflex, and triceps reflex, and positive Hoffmann reflex; the sum score (maximum four points) was defined as the UMN score for an upper limb. Similarly, exaggerated patellar reflex, exaggerated Achilles reflex, and extensor Babinski reflex constituted the UMN score for a lower limb (maximum three points). Items on muscle tonus were not included because of incomplete information. Finally, UMN sign severity was grouped into “none”, “mild,” and “severe” based on the UMN score. The upper-limb UMN score of zero corresponded to none, one and two to mild, and three and four to severe. Similarly, the lower-limb UMN score of zero was assigned to none, one to mild, and two and three to severe.

### 2.3 Central motor conduction time (CMCT)

To measure CMCT, TMS had been conducted using a standard protocol (Groppa et al., 2012). In brief, EMG recordings were obtained with surface electrodes on the first dorsal interosseous (FDI) and tibialis anterior (TA) muscle on both sides using the belly-tendon montage. The signals were amplified and filtered using a band-pass filter from 100 Hz to 3 kHz (Neuropack, Nihon Kohden Corp., Japan). Single pulse TMS was delivered to the hand and leg area of the M1, respectively, using round and double-cone coils (Magstim 200, The Magstim Co. ltd., UK). The target muscles were slightly activated voluntarily during the cortical stimulation. Foraminal electromagnetic stimulation was conducted using the same stimulator to obtain the peripheral motor conduction time; the round coil was placed over the C7 cervical spine and L5 lumbar spine. Then CMCT was calculated by subtracting the peripheral motor conduction time, which was defined as the shortest latency achieved by the foraminal stimulation, from the shortest cortical latency obtained by the cortical stimulation. Abnormality of the TMS testing was classified as either prolonged CMCT or inexcitable MEP by cortical stimulation. Prolonged CMCT of FDI was defined as 7.9 ms or longer. This was our in-house cut-off value, which is comparable with other studies (Claus, 1990, Tokimura et al., 2020). Prolonged CMCT of TA was 17.0 ms or longer (Matsumoto et al., 2010). If cortical stimulation with maximal tolerable intensity did not elicit robust MEP, then it was considered inexcitable for that limb.

### 2.4 Data analysis

Clinical and physiological data were aggregated on the disease and limb basis. The UMN signs and CMCT testing for right and left sides were analyzed together, where the right and left limbs were both counted as one limb each, i.e., each patient provided data on two upper limbs and two lower limbs for physiological parameters.

Demographic findings were compared between ALS and MSA using independent *t*-test or χ^2^ test. Differences in the UMN signs and CMCT abnormality between ALS and MSA were compared using the χ^2^ test. Relations between the CMCT value and UMN signs were explored using two-way analysis of variance (ANOVA) separately for the upper and lower limbs, where DISEASE (two levels: ALS and MSA) and UMN sign (three levels: none, mild, and severe) were between-subjects factors. Given we had no within-subject factor, repeated-measures ANOVA was not used. Height was included as a covariate. *Post-hoc* tests were conducted using the Bonferroni method as a correction for multiple comparisons. To assess influence of more general clinical status on CMCT, correlation between the Barthel Index and CMCT was tested.

To explore the influence of the disease subtype, CMCT abnormality was classified as normal or abnormal. Prolonged and inexcitable CMCT were considered abnormal; the others were normal.

Information on the LMN function could be inferred in 58 ALS patients by amplitude of compound muscle action potential (CMAP) and latency of F-wave recorded from abductor pollicis brevis (APB) of the hand and abductor hallucis (AH) of the foot. Correlations were investigated between FDI-CMCT and APB CMAP/F-latency, and TA-CMCT and AH CMAP/F-latency.

Significance was inferred for *p* ≤ 0.05. All statistical analyses were conducted using software (SPSS version 25.0; IBM Corp.).

## 3 Results

In total, medical records from 61 ALS patients (35 men and 26 women) and 61 MSA patients (40 men and 21 women) were reviewed. Their characteristics are presented in Table 1. The ALS patients were older than the MSA patients. The MSA patients were taller and had longer disease duration than the ALS patients. According to the diagnostic criteria, 11 ALS patients were classified as definite, 19 as probable, and 21 as possible. The other 10 did not meet the criteria at the time of TMS testing, but later observation confirmed that at least the criteria for possible ALS was met in all patients. Onset body parts were bulbar region in 15, upper limb in 30, and lower limb in 16 patients. Out of the 61 MSA patients, 45 were MSA-C, and 16 were MSA-P: 26 fulfilled the criteria for probable MSA and 35 for possible MSA.

**Table 1:** Demographic characteristics.

	ALS	MSA	*p*-value*
Gender (female: male)	26:35	21:40	0.352
Age at test (y)	68.7 (7.2)	62.3 (8.7)	< 0.001
Disease duration (y)	1.6 (1.2)	3.3 (2.6)	< 0.001
Body height (cm)	160.5 (8.4)	164.6 (8.4)	0.007
Barthel Index	76.4 (24.0)	78.1 (21.5)	0.703

Values are presented as mean (standard deviation).

*χ^2^ test for the gender, and *t*-tests for the others.

### 3.1 UMN signs

**Fig. 1:**
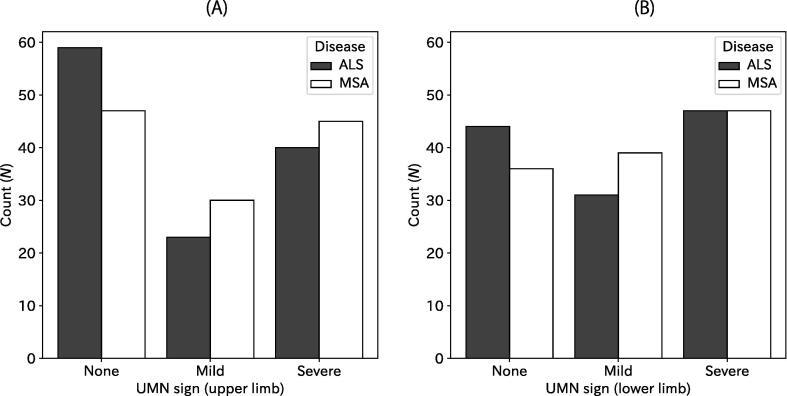
Histograms for UMN signs of the upper (A) and lower limb (B). Numbers of limbs are presented according to disease (ALS or MSA) and severity in the UMN for the upper limb (A) and lower limb (B).

Severity of the UMN sign was not significantly different between ALS and MSA. Fig. 1 shows that the numbers of limbs with no, mild, or severe UMN signs are comparable between ALS and MSA, both for the upper and lower limbs (Fig. 1; *p* = 0.276 and 0.424, χ^2^-test). More specifically, 59 upper limbs of ALS (48%) did not show UMN signs, 23 (19%) showed mild and 40 (33%) showed severe signs, whereas 47 (39%), 30 (25%), and 45 (37%) upper limbs of MSA presented, respectively with no, mild, and severe UMN signs. Similarly, 44 (36%), 31 (25%), and 47 (39%) lower limbs of ALS presented respectively with no, mild, and severe UMN signs, and 36 (30%), 39 (32%), and 47 (39) lower limbs of MSA respectively showed no, mild, and severe UMN signs.

### 3.2 Central motor conduction time (CMCT)

**Fig. 2:**
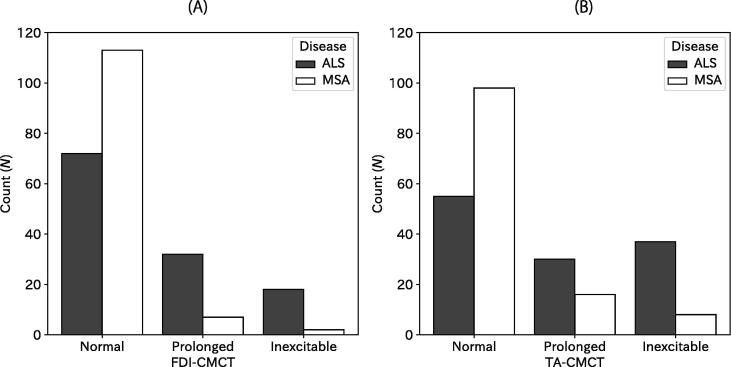
Histograms for results of CMCT. Numbers of limbs are presented according to disease (ALS or MSA) and results of the CMCT testing for the upper limb (A) and lower limb (B).

In contrast with the clinical UMN signs, CMCT was more abnormal in ALS patients than in MSA patients (Fig. 2). Specifically, prolonged FDI-CMCT was observed in 32 (26%), inexcitable in 18 (15%) ALS upper limbs, whereas seven (5%) MSA patients showed prolonged and two (2%) inexcitable FDI-CMCT. Prolonged TA-CMCT was observed in 30 (25%), and inexcitable in 37 (30%) for ALS. In MSA patients, they were observed in 16 (13%) and 8 (7%). Statistical analyses confirmed that results of the CMCT testing differed between ALS and MSA (both *p* < 0.001, χ^2^-test). Neither FDI-CMCT nor TA-CMCT were correlated with the Barthel Index significantly (*p* = 0.789 and 0.891, respectively).

### 3.3 Relation between the UMN score and CMCT

**Fig. 3:**
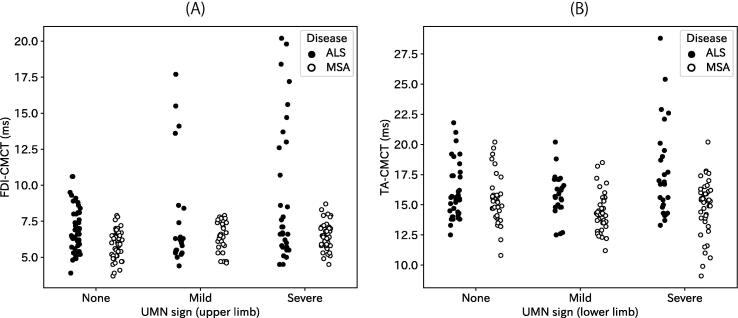
Relation between CMCT and UMN sign. CMCT values are shown according to disease (ALS and MSA) and UMN sign (none, mild, and severe). Each dot represents a limb: (A) FDI-CMCT, (B) TA-CMCT.

Fig. 3 compares CMCT between ALS and MSA according to severity in the UMN sign. ANOVA revealed significant main effects of DISEASE and UMN sign for both FDI-CMCT (DISEASE: F_1,217_ = 22.78, *p* < 0.001; UMN sign: F_2,217_ = 6.52, *p* = 0.002) and TA-CMCT (DISEASE: F_1,192_ = 36.01, *p* < 0.001; UMN sign: F_2,192_ = 4.06, *p* = 0.019). Interaction between them was significant for TA-CMCT (F_2,192_ = 7.15, *p* = 0.001), but not for FDI-CMCT (F_2,217_ = 2.46, *p* = 0.09). The covariate (height) was significant for TA (F_1,192_ = 17.55, *p* < 0.001), but not for FDI (F_1,217_ = 0.84, *p* = 0.36). *Post-hoc* analyses were conducted for each disease. With FDI-CMCT of ALS, a one-way ANOVA revealed a significant main effect of UMN sign (F_2,101_ = 3.63, *p* = 0.030), and patients with severe UMN sign had longer CMCT than those without UMN sign (*p* = 0.025, *t*-test with Bonferroni correction). Similarly, TA-CMCT of ALS was affected significantly by the UMN sign (F_2,82_ = 3.82, *p* = 0.026), but the difference among degrees of UMN sign were only marginal (*p* = 0.051 between patients with mild UMN sign and those with severe sign, and *p* = 0.058 between patients with severe UMN sign and those without, *t*-test with Bonferroni correction). With FDI-CMCT of MSA, the ANOVA revealed a significant main effect of UMN sign (F_2,117_ = 4.80, *p* = 0.010). Findings indicate that MSA patients with severe UMN sign had longer CMCT than those without UMN sign (*p* = 0.018, *t*-test with Bonferroni correction). TA-CMCT did not differ across different degrees of the UMN sign in MSA (F_2,111_ = 1.92, *p* = 0.152).

### 3.4 Disease subtype

In the ALS patients, onset body-part affected the CMCT results. For the bulbar-onset ALS patients, abnormal CMCT was less likely for both FDI and TA (Table 2). Also, patients with lower-limb onset were less likely to have normal TA-CMCT (*p* = 0.001 (FDI) and < 0.001 (TA), χ^2^-test). Differences in the MSA subtype had no influence on CMCT test results (*p* = 0.776 (FDI) and 0.879 (TA), χ^2^-test, Table 3).

**Table 2:** CMCT results in different onset body part in ALS.

(A) FDI-CMCT and onset body part in ALS			
Onset	Normal	Abnormal	Total
Bulbar	26	4	30
Upper limb	27	33	60
Lower limb	13	19	32
Total	66	56	122

(B) TA-CMCT and onset body part in ALS			
Onset	Normal	Abnormal	Total
Bulbar	21	9	30
Upper limb	29	31	60
Lower limb	5	27	32
Total	55	67	122

**Table 3:** CMCT results in different subtypes of MSA.

(A) FDI-CMCT and subtype in MSA			
Subtype	Normal	Abnormal	Total
MSA-C	83	7	90
MSA-P	30	2	32
Total	113	9	122

(B) TA-CMCT and subtype in MSA			
Subtype	Normal	Abnormal	Total
MSA-C	72	18	90
MSA-P	26	6	32
Total	98	24	122

### 3.5 Influence of LMN dysfunction on CMCT

Correlation analysis between CMAP or F-latency and CMCT turned out to be insignificant. The *p*-values were 0.879 (FDI-CMCT and APB-CMAP), 0.955 (FDI-CMCT and APB F-latency), 0.574 (TA-CMCT and AH-CMAP), and 0.644 (TA-CMCT and AH F-latency).

## 4 Discussion

We explored relations between clinical UMN signs and CMCT in ALS and MSA. The main findings were that (1) ALS and MSA patients showed clinical UMN signs with similar frequency and severity, and that (2) CMCT was more abnormal in ALS than in MSA. The onset body part was associated with CMCT abnormality in ALS, but MSA subtype was not.

### 4.1 Clinical signs

Results show that profiles of the UMN signs were similar in ALS and MSA, as indexed by the UMN score. Evidence shows that the UMN signs are observed in around 40–70% of ALS patients (Gubbay et al., 1985, Zoccolella et al., 2006), and that MSA patients presented with Babinski sign in 7–59% and hyper-reflexia in 13–73% of cases in different countries (Köllensperger et al., 2010). Our results are in line with these reported studies of the literature, indicating that the population represented common phenotypes of ALS and MSA.

### 4.2 CMCT testing and its implications for pathophysiology of ALS and MSA

In stark contrast to the clinical signs, the CMCT testing results were different between the two diseases. Indeed, earlier studies failed to document degeneration of the UMN in MSA using physiological or neuroimaging measures (Abbruzzese et al., 1997, Abele et al., 2000, Sobue et al., 1992). Sobue et al. evaluated CMCT and spinal-cord pathology, revealing normal CMCT accompanied by small-fibre depletion in MSA (Sobue et al., 1992), with relative preservation of large fibres in the corticospinal tract. In ALS patients, abnormality of the CMCT testing has been established (Civardi et al., 2020, Groppa et al., 2012, Ugawa et al., 1988). It can even “unmask” the UMN signs that were not readily apparent from clinical examination alone (Tokimura et al., 2020, Zoccolella et al., 2020).

Why are the UMN signs and results of the CMCT testing incongruent between ALS and MSA? One might consider involvement of the LMN dysfunction. With foraminal stimulation, the calculated CMCT includes conduction between the motoneuron and the foramen, and it is possible that pathology here contributes to the longer CMCT in ALS. Alternatively the difference in CMCT could reflect different pathologies of the pyramidal tract in ALS and MSA. Large myelinated fibres comprising the direct cortico-motoneuron system are considered the main target of degeneration in ALS (Lemon, 2021, Smith, 1960, Sobue et al., 1987), whereas selective loss of the small-diameter myelinated fibres of pyramidal tract were noticed in MSA (Sobue et al., 1987). Because CMCT is believed to be more representative of large-diameter than small-diameter fibres with fast conduction velocity in the pyramidal tract (Tang et al., 2020, Udupa and Chen, 2013), such pathological difference would be a plausible reason for different CMCT between the two despite similar clinical features. What remains to be elucidated is the pathological confirmation of this notion.

### 4.3 Clinico-physiological correlation in ALS

Interestingly, CMCT was prolonged or inexcitable in ALS patients even without UMN signs, especially in the upper limb. Such discrepancy can be resolved by incorporating at least two factors. The first is the fact that very severe lower motor neuron (LMN) signs sometimes conceal co-existing UMN signs. The most conspicuous example would be the primary progressive atrophy, a subtype of ALS, for which autopsy often reveals degeneration in the corticospinal tract in spite of absent UMN signs throughout the clinical course (Ince et al., 2003). Such masking does not occur in MSA. The other factor is the characteristic distribution of degeneration in ALS (Swash, 2012). Among the motor descending pathways, the corticospinal tract is adversely affected by far with the most severe damage. Abnormality in the corticospinal tract would cause abnormal CMCT testing, but other preserved tracts might prevent clinical UMN signs from manifesting. This presents a sharp contrast to other myelopathies that usually present with the typical UMN signs. Degeneration of spinal interneurons might also play a role (Swash, 2012).

In comparison with FDI-CMCT, TA-CMCT was not found to be significantly different across the severity of the UMN signs. One possible explanation for this finding is that CMCT estimated by the peripheral motor conduction time with the foraminal stimulation overestimates the central component. Prolonged TA-CMCT might be explained by the LMN signs of the leg. Another consideration would be the composition of the corticospinal tract. Evolutionally speaking, the descending pathway in the hand contains more direct corticospinal fibres than the leg (Lemon, 2008). Such differentiation might underlie the current observations.

Results show that ALS patients with different body parts as the onset site presented with different CMCT findings. It is quite straightforward that bulbar-onset ALS patients showed CMCT abnormality less frequently because the test was performed with the upper and lower limb. Objective tests must be developed to elucidate UMN degeneration in the cortico-bulbar tract.

Because CMCT measurements with the foraminal stimulation are contaminated by conduction from the motoneuron to the foramen, we looked for a correlation between the nerve conduction findings and CMCT in the ALS patients. There was none, but unfortunately the nerve conduction studies were not performed on the same muscles as CMCT. This issue requires further confirmation in future studies.

### 4.4 Clinical usefulness

CMCT can increase the chance of detecting degeneration of the corticospinal tract in the absence of clinical UMN signs. A neuroimaging study partially confirmed this notion by showing correlation between CMCT and parameters of diffusion tensor imaging associated with white matter degeneration (Ogura et al., 2022). Abnormal CMCTs can be regarded as an objective marker to diagnose ALS without clinically apparent UMN signs. Furthermore, a recent article proposed inexcitable M1 by TMS as a good prognostic marker (Dharmadasa et al., 2021). This issue warrants further investigation. More specifically, combining paired-pulse TMS techniques and other advanced methods including neuroimaging technique can elucidate mechanisms in the UMN pathology of ALS and MSA.

### 4.5 Limitations

This study has several limitations mainly originating from the retrospective nature. Some demographic factors are different between ALS and MSA: The ALS patients were older than the MSA patients; the MSA patients were taller and had longer disease duration. We have no relevant neuroimaging or neuropathological results to confirm fibre-type difference in degeneration of the corticospinal tract. Other TMS parameters including motor thresholds and MEP amplitude could not be extracted in a comprehensive manner. Also, CMCT calculation using the F-wave latency was not available in the present study. These issues must be addressed in future prospective studies.

## 5 Conclusions

In conclusion, CMCT testing revealed differential abnormality between ALS and MSA with similar profiles of the clinical UMN signs. Pathological differences might underlie the physiological distinction. Future research including investigations of autopsy findings and longitudinal observations is warranted.

## Funding

This work was supported by JSPS KAKENHI Grant No JP 21K07454 (YS) and 22K07534 (MH), and by a grant from the 10.13039/100016163Kanae Foundation for the Promotion of Medical Science to YS. Part of this paper was submitted as a thesis by JO to the 10.13039/100005688Graduate School of Medicine, the University of Tokyo.

## Declaration of Competing Interest

The authors declare that they have no known competing financial interests or personal relationships that could have appeared to influence the work reported in this paper.
